# No link between piriform cortex subregion resection and seizure freedom in two cohorts with temporal lobe epilepsy

**DOI:** 10.1007/s00415-026-13850-w

**Published:** 2026-05-22

**Authors:** Felix Zahnert, Florian Pfanzelt, Katja Menzler, Lena Habermehl, Boris Keil, Lars Timmermann, André Kemmling, Alexander Grote, Christopher Nimsky, Susanne Knake, Marcus Belke

**Affiliations:** 1 School of Medicine, Department for Neurology, Philipps-Universität Marburg, Baldingerstr, 35043 Marburg, Germany; 2 Center for Mind, Brain and Behavior (CMBB), Philipps-Universität Marburg, Marburg, Germany; 3 Institute of Medical Physics and Radiation Protection, Technische Hochschule Mittelhessen, Giessen, Germany; 4 School of Medicine, Department for Neuroradiology, Philipps-Universität Marburg, Marburg, Germany; 5 School of Medicine, Department for Neurosurgery, Philipps-Universität Marburg, Marburg, Germany; 6 Goethe-University Frankfurt, LOEWE Center for Personalized Translational Epilepsy Research (Cepter), Frankfurt Am Main, Germany

**Keywords:** Epilepsy, Epilepsy surgery, Piriform cortex, Temporal lobe epilepsy, Volumetry, Voxel-based lesion-outcome mapping

## Abstract

**Background:**

Postoperative seizure freedom in temporal lobe epilepsy (TLE) has been linked to the extent of piriform cortex resection. Earlier work used manual piriform cortex segmentations truncated at the limen insulae and overlapping the amygdala. We re-evaluated this association using an investigator-independent, connectivity-based segmentation.

**Methods:**

Patients with TLE who underwent mesiotemporal epilepsy surgery at a single center with > 1-year postoperative follow-up were included retrospectively (discovery cohort). Postoperative images and connectivity-defined piriform cortex subregions were registered to preoperative T1-weighted scans. Additional manual piriform cortex segmentation was performed for comparison. The extent of resection within temporal lobe subregions was compared between seizure-free (ILAE I) and non-seizure-free patients. Results were validated using a large independent cohort. For additional validation, voxel-based lesion-outcome mapping was performed using logistic regression.

**Results:**

Twenty-eight patients (15 female) with TLE of heterogeneous etiologies were included in the discovery cohort. No significant association between ILAE outcome and the extent of resection within manually or automatically defined piriform cortex was observed. Extensive analyses in the validation cohort (*n* = 305, 205 with hippocampal sclerosis) confirmed this result. Additional voxel-based lesion-outcome mapping (*n* = 305) showed no significant associations with ILAE I outcome. Resected proportions within other mesiotemporal regions were not associated with ILAE I outcome.

**Conclusion:**

No association between ILAE outcome and the resected proportion of specific mesiotemporal structures was detected. This result was robust across cohorts, piriform cortex definitions and analytical approaches. These results differ from previous reports and suggest that piriform cortex resection should be considered on an individualized basis rather than routinely incorporated into temporal lobe resections.

**Supplementary Information:**

The online version contains supplementary material available at 10.1007/s00415-026-13850-w.

## Introduction

Approximately one-third of the patients with focal epilepsy are resistant to anti-seizure medications [1]. For these patients epilepsy surgery should be considered as an effective treatment option [2]. Patients with temporal lobe epilepsy (TLE) are commonly offered standard anterior temporal lobe resections (ATLR) or less extensive selective amygdalo–hippocampectomies (SAHE). The rate of long-term postoperative seizure freedom is reported around 50–70% [3–9], with a larger proportion of seizure free patients after ATLR [10, 11]. Recurring postoperative seizures might sometimes be attributed to preoperative lack of understanding of the individual organization of the patient’s epileptogenic network, which can extend into, e.g. orbitofrontal or cingulate cortices [12].

In addition, in focal epilepsies, the small piriform cortices seem to show a high likelihood for involvement in epileptogenic networks [13], [14, 15]. This three-layered primary olfactory cortex is highly susceptible to kindling and chemoconvulsants in rodents and monkeys [16, 17]. In human EEG-fMRI experiments, the piriform cortex has been shown to be functionally activated during epileptiform discharges [18]. Its involvement in epileptogenic networks is further supported by a decrease in volume [19, 20] and an increase in structural network integration in patients with drug resistance and longer durations of epilepsy [21]. The extent of resections of the ipsilateral piriform cortex seemed to be associated with a favorable outcome after ATLR [22]. This finding has been reproduced in retrospective cohorts by two independent groups [23–25], although this was not the case in pediatric patients [26]. An intracranial EEG study could not confirm common piriform cortex involvement in temporal lobe seizures (N. P. [27]). Functionally, the piriform cortex plays a role in odor identification and discrimination [28, 29], odor-related memory [30–32], and spatial navigation [33, 34]. Its resection did not negatively impact postoperative cognitive outcomes [22, 24], and it did not increase the occurrence of postoperative psychosis, depression or anxiety in previous retrospective studies [22], while its proximity to the middle cerebral artery requires caution [15].

In previous studies, the delineation of the piriform cortex relied on manual segmentation. This followed a pragmatic protocol involving also cortical parts of the superficial amygdala for a lack of visual discriminability of the mesial, posterior and rostral boundaries of the piriform cortex on T1 images [19]. Previous retrospective epilepsy surgical studies acknowledge the difficulty to delineate the extent of the piriform cortex on structural MRI [26]. A recent connectivity-based parcellation approach has shown that the piriform cortex and the cortical amygdala seem to be distinct, and that, contrary to the pragmatic manual protocol, the piriform cortex extends anteriorly beyond the limen insulae [35].This is in line with histological findings [36].

In the present study we investigated the association of resections of connectionally defined piriform cortex subregions (one frontal and two temporal subregions) with an ILAE I outcome after mesiotemporal epilepsy surgery. Our hypothesis was that resections of these subregions, whose overall extent seems more specific to the piriform cortex and in line with histological studies [36, 37], might show stronger associations with seizure freedom than analysis of resection volumes of manually defined, whole piriform cortices. To ensure the reliability of our results, two independent datasets comprising an overall 333 patients with temporal lobe epilepsy surgery were analyzed, and additional voxel-based lesion outcome mapping was conducted for further validation.

## Methods

### Patients

All patients who had received temporal lobe epilepsy surgery at the Epilepsy Center Hesse (University Hospital Marburg) between 2009 and 2022 were identified retrospectively. The inclusion criteria for the present study were (1) available pre- and postoperative 3D-T1 scans without contrast agents, scanned at least three months after surgery; (2) available clinical information on ILAE outcomes after surgery with a minimum follow-up of one year; (3) resection of mesiotemporal structures via either SAHE, ATLR or tailored lesionectomies; (4) a minimum age of 18 years at the time of the preoperative scan.

Exclusion criteria were (1) exclusively temporolateral or temporobasal resections without involvement of the mesial temporal lobes; (2) age < 18 years; (3) use of contrast agent on either pre- or postoperative image.

Surgical outcomes of some patients of this cohort (16/28 patients) have been published before [21].

In addition, the ‘Imaging Database for Epilepsy and Surgery’ (IDEAS) dataset [38] was leveraged to validate our results. Here, all patients who had received either ATLR or temporal lobe lesionectomy due to temporal lobe epilepsy were included if postoperative ILAE outcomes after ≥ 1-year were available. Patients were excluded if no resection masks were available or if neuroimaging data quality did not allow image processing. The dataset was split into one large homogeneous group of patients with hippocampal sclerosis who had received ATLR, as well as another large group comprised of patients with etiologies other than HS who had received either lesionectomy or ATLR.

The study was approved by the Institutional Review Board of the University of Marburg School of Medicine (IRB00011440) in accordance with the Declaration of Helsinki. The requirement for informed consent was waived for the use of anonymized retrospective data in this study (approval number RS22/57).

### Image acquisition

Due to the retrospective study design, acquisition protocols and scanners varied. Four scanners had been used for acquisition of pre- and postoperative 3D T1-weighed data in the discovery cohort (Marburg): (1) 3 T Siemens Trio, (2) 3 T Siemens Verio (3) 1.5 T Siemens Avanto (3), 1.5 T Siemens Espree (Siemens, Erlangen, Germany). The echo time ranged from 2.26 to 3.14 ms, the inversion time was 0.9–1.1 s, the repetition time was 1.9–2.1 s and the flip angle was 9–15°. Voxel resolution was 1 mm isotropic in all cases, except for one scan with 0.9 mm isotropic resolution. Detailed acquisition parameters for the 305 patients from the validation (IDEAS) dataset are reported in the dataset publication [39].

### Preprocessing of structural images

Freesurfer 7.4.1 was used for bias field correction, surface extraction, intensity normalization and cortical and subcortical parcellation (recon-all pipeline) [40–42]. Subfields within the hippocampus and the amygdala were segmented using the tools available in the Freesurfer 7.4.1 package [43, 44].

### Registration and brain shift correction

Coregistration of pre- and postoperative structural images for each subject was performed using linear and non-linear transformations implemented in the ANTS software package [45, 46]. The non-linear registration step provided correction for brain shift and was visually inspected in every subject. Preoperative images were also registered to the MNI152 template with ANTS, enabling transformation of the piriform cortex ROI from standard to native preoperative space. All subsequent computations were carried out in the individual preoperative space.

### Semi-automated delineation of the resection zone

Mean normalized T1-intensities were extracted for each parcel of the preoperative brain according to the Desikan parcellation [47] and subcortical segmentation as obtained from Freesurfer. These were then compared to the aligned postoperative brain, and cut-off values for postoperative loss of T1 intensity in white matter and grey matter were found. Voxels with decreases in T1-intensity exceeding this threshold were labeled as resected. All resulting individual binary resection masks were then inspected and manually corrected where necessary (author FZ).

### Acquisition of piriform cortex regions of interest

**Fig. 1:**
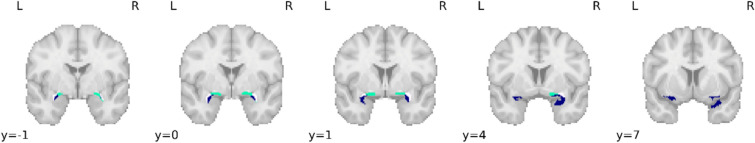
Piriform cortex subregions visualized on coronal sections of the MNI152 brain. Cyan = frontal piriform cortex, blue = anterior/temporal piriform cortex, white = dorsotemporal piriform cortex

Piriform cortex subregions had been defined and distinguished from neighboring amygdala and insula based on their structural connectivity in a previous connectivity-based parcellation study in healthy subjects [[35], https://github.com/Zahnert/piriform_cbp/tree/main]. From the available solutions, bilateral 7-cluster solutions were selected, as both parcellations had produced similar three piriform subdivisions (Fig. 1) and had shown high consistency across a group of healthy participants [35]. Within these bilateral parcellations, three homologous regions corresponded to piriform cortex subregions, respectively, while the remaining parcels were either parts of the amygdala or insula. These three regions of interest (here termed frontal, anterior, and dorsotemporal PC) were registered from MNI152 space to native structural space for further analyses. Accurate registration of piriform cortex ROI was verified by visual inspection.

For comparison, piriform cortices ipsilateral to the resection zone were also manually delineated on preoperative 3D T1-weighted scans in accordance with a published protocol [19, 22].

### Computation of regional extent of resections

The overlap of temporal lobe regions with the resection zone in percent was computed in each patient. Hippocampal subfield resections were investigated using the hippocampal subfield segmentation algorithm implemented in Freesurfer version 7.4.1.

### Validation

The recently published IDEAS dataset [38] was leveraged to validate the results obtained from our small discovery cohort. Masked resection zones were already available in preoperative space, while postoperative T1 scans were unavailable. Therefore, in this cohort resection zones could not be delineated using our above-mentioned method. However, the automated segmentation methodology employed by the authors of the IDEAS dataset was highly similar to that reported here [38].

Preoperative images were registered to MNI152 space to allow for resampling of piriform cortex and subregion labels to native structural space. Due to the size of this sample, no manual delineation of the piriform cortex was conducted here. All further analyses were the same as described for the main cohort.

### Statistics

#### Outcome of interest

The main outcome analyzed in this study was postoperative seizure control, as classified by the ILAE[48]:

ILAE I: Completely free from seizures and auras.

ILAE II: persisting auras, but no other seizure types.

ILAE III: one to three seizure days/year.

ILAE IV: four seizure days/year to 50% reduction of baseline seizures.

ILAE V: < 50% reduction in yearly seizure days to 100% increase in seizure days.

ILAE VI: > 100% increase in baseline seizure days/year.

ILAE III – VI: all ± auras.

In this study, we investigated the binary outcome of complete seizure freedom (ILAE I) vs persistent seizures of any quantity and kind (ILAE II–VI).

#### Volumetric analysis

We compared the percentage extent of regional resections between patients with ILAE class I outcomes and those with ILAE classes II–VI outcomes using non-parametric permutation *t* tests (100,000 permutations). *p* values across regions were adjusted for multiple comparisons using the Benjamini–Hochberg false discovery rate (FDR) procedure (*q* = 0.05).

In the validation cohort, the above-mentioned volumetric analyses were conducted separately in two subgroups: one with HS who had received ATLR, and one without HS.

#### Voxel-based lesion outcome mapping

In addition, voxel-based lesion-outcome-mapping was conducted [similar to [49]]. Patients with hippocampal sclerosis and epilepsies of other etiologies were pooled for this analysis to maximize statistical power, while lesion type was included as a covariate. Each patient’s binary resection mask was registered to MNI152 2009a nonlinear asymmetric space. Voxel-wise logistic regression was conducted, where the predictor at each voxel was whether that voxel was resected (0/1), and the dependent variable was seizure freedom (ILAE I vs II–VI, coded as 0/1). Covariates included in the design matrix were sex, overall resection volume, the type of surgery (ATLR vs. lesionectomy) and the type of lesion (hippocampal sclerosis vs. other lesions). Correction for the family-wise error was conducted at the cluster level. The voxelwise cluster-forming threshold was set at p uncorrected = 0.001, and cluster significance (based on cluster mass) was assessed using permutation testing (*n* = 5000). This analysis was conducted in left (*n* = 170) and right (*n* = 135) TLE separately. Voxelwise beta coefficients for ILAE-I outcomes from logistic regression were converted to odds-ratios (OR = e^β) and visualized on the MNI152 brain.

To increase specificity of the analysis, a second approach to lesion-outcome-mapping was explored. Here, only patients with HS and ATLR (n = 205) were included. Registration of resection masks to the symmetric MNI ICBM 2009a Nonlinear Symmetric template [freesurfer EasyReg [50]] allowed for subsequent flipping of right resection masks onto the left hemisphere. This was done to achieve a sufficient sample size (by pooling patients with left and right resections for logistic regression). Logistic regression with cluster-wise control of the family-wise error rate was then conducted within the left hemisphere including all 205 patients with HS.

Statistical testing was performed using the following python libraries: scikit-learn (https://scikit-learn.org/stable/about.html) and scipy [51]). Nilearn (https://nilearn.github.io/stable/index.html) and seaborn [52] were used for visualization.

## Results

### Patient characteristics

#### Discovery cohort (Marburg)

Thirty-seven patients who had received epilepsy surgery within the temporal lobe and who had adequate pre- and postoperative T1 images without contrast agent were identified. Six patients were excluded from this cohort due to exclusively temporobasal or temporolateral surgeries without involvement of the mesial temporal lobe, as well as one pediatric patient (aged 10 years). Further two patients with follow-up periods < 1 year were excluded.

Therefore, 28 patients who had received mesiotemporal epilepsy surgery were included in the study (15 female). Twelve patients (42.9%) were seizure free (ILAE I at last follow-up). Clinical characteristics of this cohort can be found in Table 1.

**Table 1:** Characteristics of the discovery (Marburg) and validation (IDEAS) datasets

a) Discovery cohort (*n* = 28)			
Characteristics	Overall *n* (%)	ILAE I, *n* (%)	ILAE II–VI, *n* (%)
Sex			
Male	13 (46.4)	6 (50)	7 (43.4)
Female	15 (53.6)	6 (50)	9 (56.3)
Laterality of TLE			
Left	18 (64.3)	7 (58.3)	11 (68.8)
Right	10 (35.7)	5 (41.7)	5 (31.3)
	Overall, M (SD)	ILAE I, M (SD)	ILAE II–VI, M (SD)
Age of onset in years	22 (18)	18.7 (19.3)	24.5 (17.1)
Duration of epilepsy at t(OP) in years	16.2 (14.2)	18.3 (15.1)	14.7 (13.7)
Postoperative follow-up in years	4.4 (1.5)	3.9(1.5)	4.7 (1.4)
	Overall *n* (%)	ILAE I, *n* (%)	ILAE II–VI, *n* (%)
Surgical procedure			
SAHE	22 (78.6)	8 (66.7)	14 (87.5)
ATL	3 (10.7)	2 (16.7)	1 (6.3)
Tailored	3 (10.7)	2 (16.7)	1 (6.3)
Lesion (clinical histopathology)			
Hippocampal sclerosis	12 (42.9)	4 (33.3)	8 (50)
Cavernoma	1 (3.6)	–	1 (6.25)
LEAT	3 (10.7)	3 (25)	–
Diffuse neuronal heterotopia	3 (10.7)	2 (16.7)	1 (6.25)
Astrocytoma WHO°II	1 (3.6)	–	1 (6.3)
Unclear	8 (28.6)	3 (25)	5 (31.3)
b) Validation cohort (IDEAS dataset, *n* = 305)			
1. Cohort with HS (*n* = 205)			
Characteristics	Overall *n* (%)	ILAE I, *n* (%)	ILAE II-VI, *n* (%)
Sex			
Male	80 (39)	46 (38)	34 (40.5)
Female	125 (61)	75 (62)	50 (59.5)
Laterality of TLE			
Left	118 (57.6)	69 (57)	49 (58.3)
Right	87 (42.4)	52 (43)	35 (41.7)
Surgical procedure			
ATLR	205 (100)	121 (100)	84 (100)
2. Cohort without HS (*n* = 100)			
Characteristics	Overall *n* (%)	ILAE I, *n* (%)	ILAE II–VI, *n* (%)
Sex			
Male	53 (53)	36 (53.7)	17 (51.5)
Female	47 (47)	31 (46.3)	16 (48.5)
Laterality of TLE			
Left	52 (52)	36 (53.7)	16 (48.5)
Right	48 (48)	31 (46.3)	17 (51.5)
Surgical procedure			
ATLR	74 (74)	47 (70.1)	27 (81.8)
Lesionectomy	26 (26)	20 (29.9)	6 (18.2)

*LEAT* long-term epilepsy-associated tumors, *ATLR* anterior temporal lobe resection, *HS* hippocampal sclerosis

#### Validation cohort (IDEAS dataset)

The validation dataset consisted of one homogenous cohort of patients with hippocampal sclerosis who had received standard ATLR (n = 205), as well as one cohort of 100 patients who had received either ATLR or lesionectomies and who had etiologies of TLE other than HS. In the HS-subgroup, 7/212 initially identified patients had been dropped due to incomplete data and failed freesurfer reconstructions. Characteristics of the validation dataset are reported in Table 1.

**Fig. 2:**
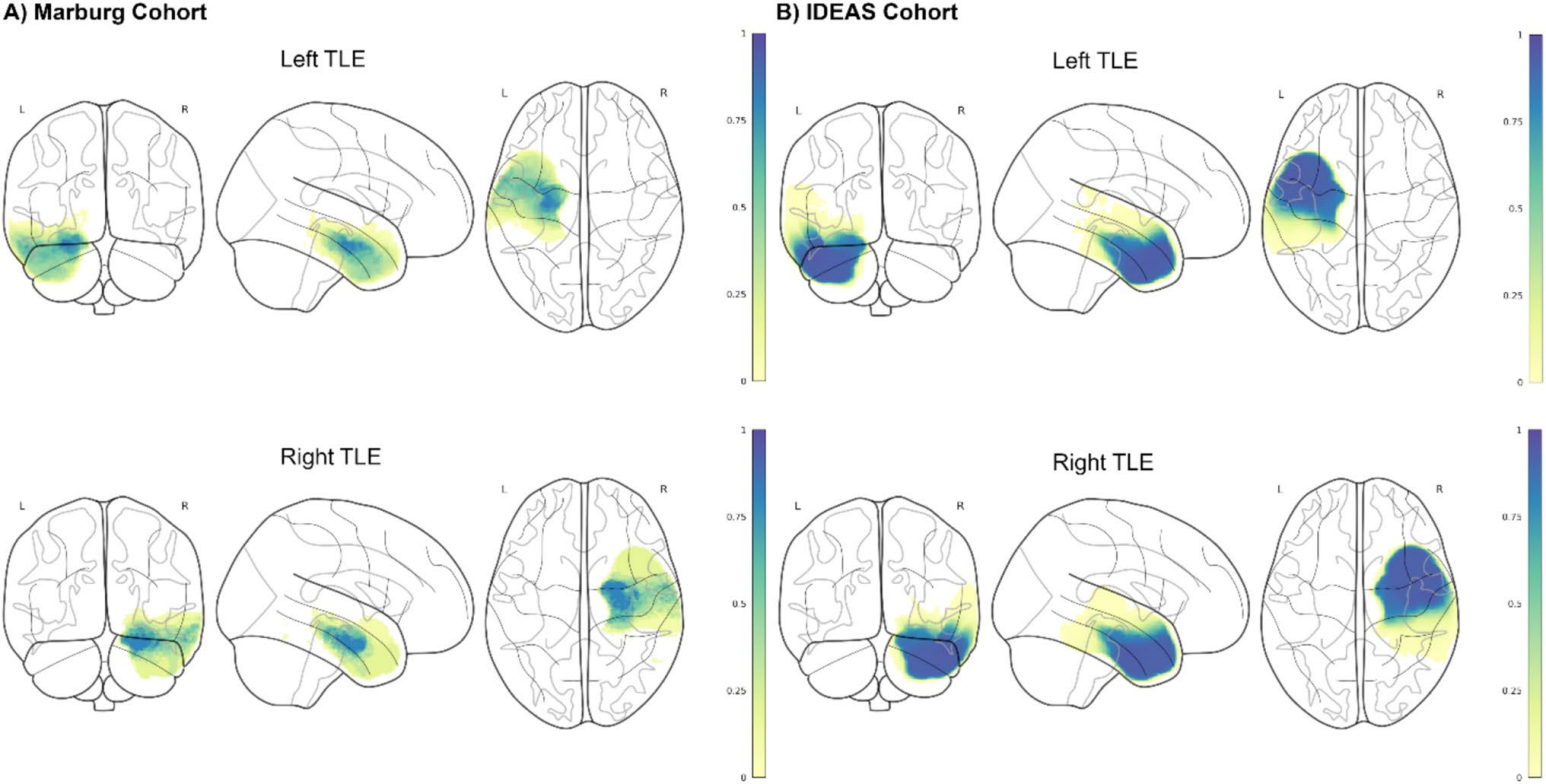
Overview on resection zones in **A** the discovery (Marburg) cohort and **B** the validation cohort (IDEAS dataset). The background image is a glass brain in MNI152 space. The colorscale represents the proportion of subjects in whom the respective voxel has been resected (0–1, where e.g. 0.5 indicates resection in 50%)

Here, ILAE I outcomes were achieved in 121/205 (59%) patients with HS and in 67/100 patients with TLE of etiologies other than HS. Figure 2 provides an overview over resection volumes across patients.

### Volumetric results in the discovery cohort (Marburg)

**Fig. 3:**
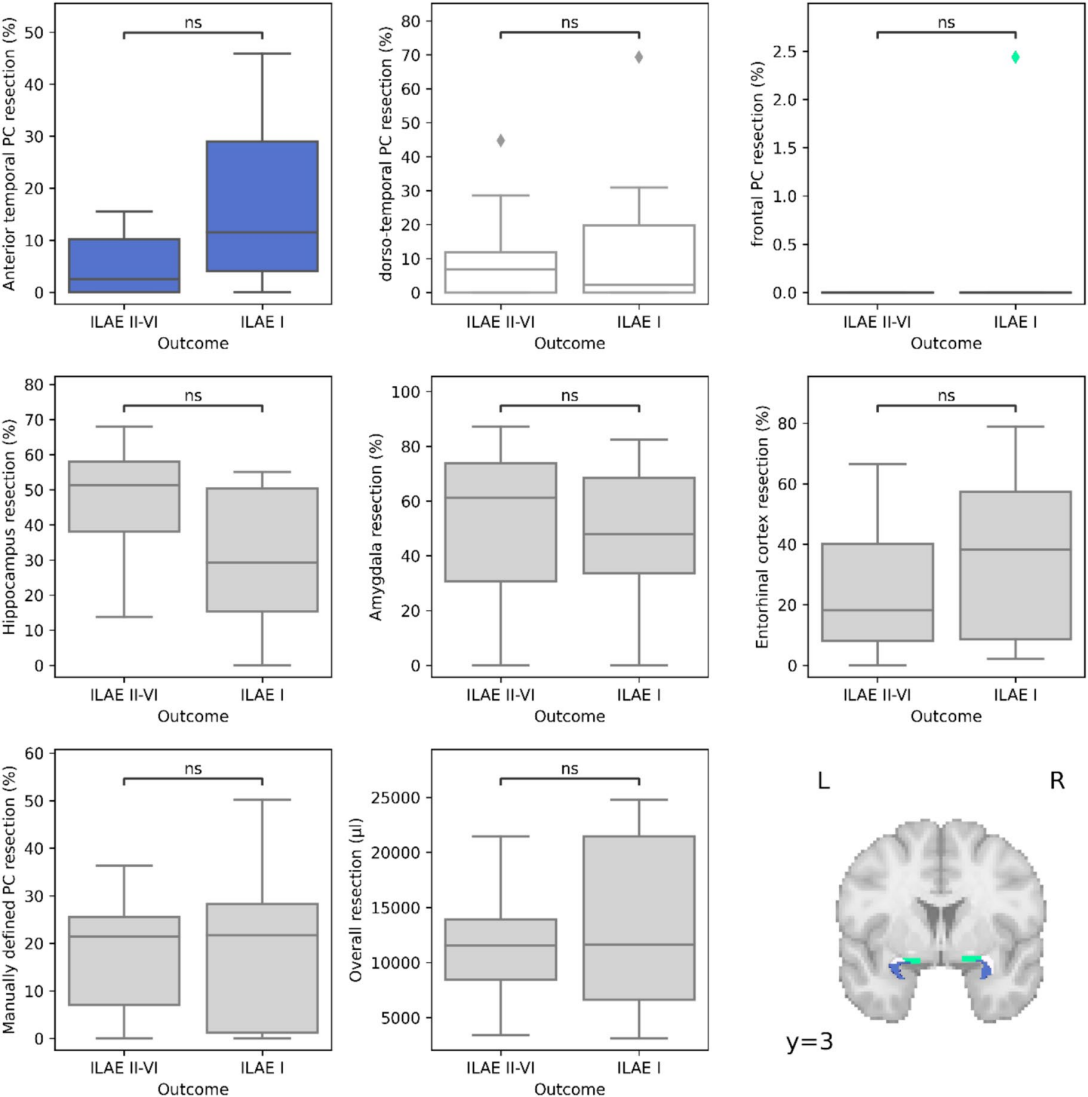
Boxplots depicting volumetric associations between subregion resections and ILAE I outcomes. Bottom right image depicts piriform subregions corresponding to top row analyses, color coded. *ns* not significant after FDR correction for 8 comparisons. *PC* piriform cortex

No significant association between the extent of subregional resections and ILAE I outcomes was detected in the discovery cohort (Fig. 3). The largest effect size was observed in the anterior portion of the piriform cortex (*t* = 2.49, *d* = 1.07, *p* = 0.0884; FDR corrected for eight comparisons). The resection of the manually defined piriform cortex, delineated in line with a previously published protocol [22], showed no association with ILAE outcomes (*t* = 0.24, *d* = 0.098, *p* = 0.808).

A trend for a negative association between hippocampal resections and ILAE I outcomes was observed (*t* = − 2.45, *d* = − 0.98, *p* = 0.0884; FDR corrected for eight comparisons).

#### Hippocampal subregion analysis

We investigated hippocampal subregional contributions to the observed trend for a negative association of resections with ILAE outcomes. Here, we found significant negative associations of resections within the hippocampal body and tail with postoperative ILAE I outcomes as the drivers of the effects observed in the main analysis (Table S1).

### Validation analysis (IDEAS dataset)

To validate our results, data from the recently published IDEAS cohort (*n* = 305 patients) [38] were analyzed.

#### Volumetric results

Volumetric analysis of piriform cortex resections was restricted to the data-driven parcellations available in standard space. Figures S5 shows registered piriform cortex labels in a representative subject.

##### Subgroup with hippocampal sclerosis

In the cohort of patients with HS who had received ATLR (*n* = 205), no associations between piriform or hippocampal resections and ILAE 1 outcomes were observed (Table 2). Hippocampal resections were not associated with ILAE II–VI outcomes in this cohort. Results remained unchanged in a subgroup analysis with a constant, identical follow-up period of 3 years (*n* = 172, Table S4). The extent of resection within subfields of the hippocampus and amygdala was not associated with postoperative outcomes (Table S3).

**Table 2:** No significant association between regional resections and ILAE I outcomes in 205 patients from the IDEAS dataset with hippocampal sclerosis and ATLR (two-tailed independent samples t-test)

	Resection ILAE I (*n* = 121) % (Median [IQR])	Resection ILAE II-IV (*n* = 84) % (Median [IQR])	T-stat	Cohen’s *d*	*p* (FDR)
Frontal piriform cortex	0 [0–0]	0 [0–0]	0.36	0.05	0.93
Dorsotemporal piriform cortex	17 [3–39.2]	23.9 [3–42.4]	− 1.39	− 0.2	0.66
Anterior piriform cortex	28.3[16.8–52.9]	30.5 [20.2–54]	− 0.68	− 0.1	0.66
Whole piriform cortex	24.8 [13–43]	24.9 [18.2–44.3]	− 0.1	− 0.14	0.66
Hippocampus	56.2 [48.6–61.3]	56.4 [46.6–62.3]	− 0.1	− 0.01	0.93
Entorhinal Cortex	96.5 [90.9–99]	96.3 [93.4–98.6]	− 0.75	− 0.1	0.66
Amygdala	82.7 [68–88.7]	81.9 [68–89.3]	− 0.9	− 0.12	0.66
	Resection volume ILAE I (µl), Median [IQR]	Resection volume ILAE II–IV (µl), Median[ IQR]	T-stat	Cohen’s *d*	*p* (FDR)
Overall resection	33,403 [28,679–37,694]	35,343 [30,030–39,403]	− 1.62	− 0.23	0.66

##### Subgroup without hippocampal sclerosis

For further validation, 100 additional patients from this dataset who had received ATLR or temporal lobe lesionectomies (including SAHE) and who had etiologies of TLE other than HS were analyzed. Again, no association between the extent of temporal lobe subregion resections and seizure freedom was observed (Supplementary Table S2). This was also the case in the analysis of subfields of the hippocampus and the amygdala (Table S4).

#### Voxel-based lesion-outcome mapping

**Fig. 4:**
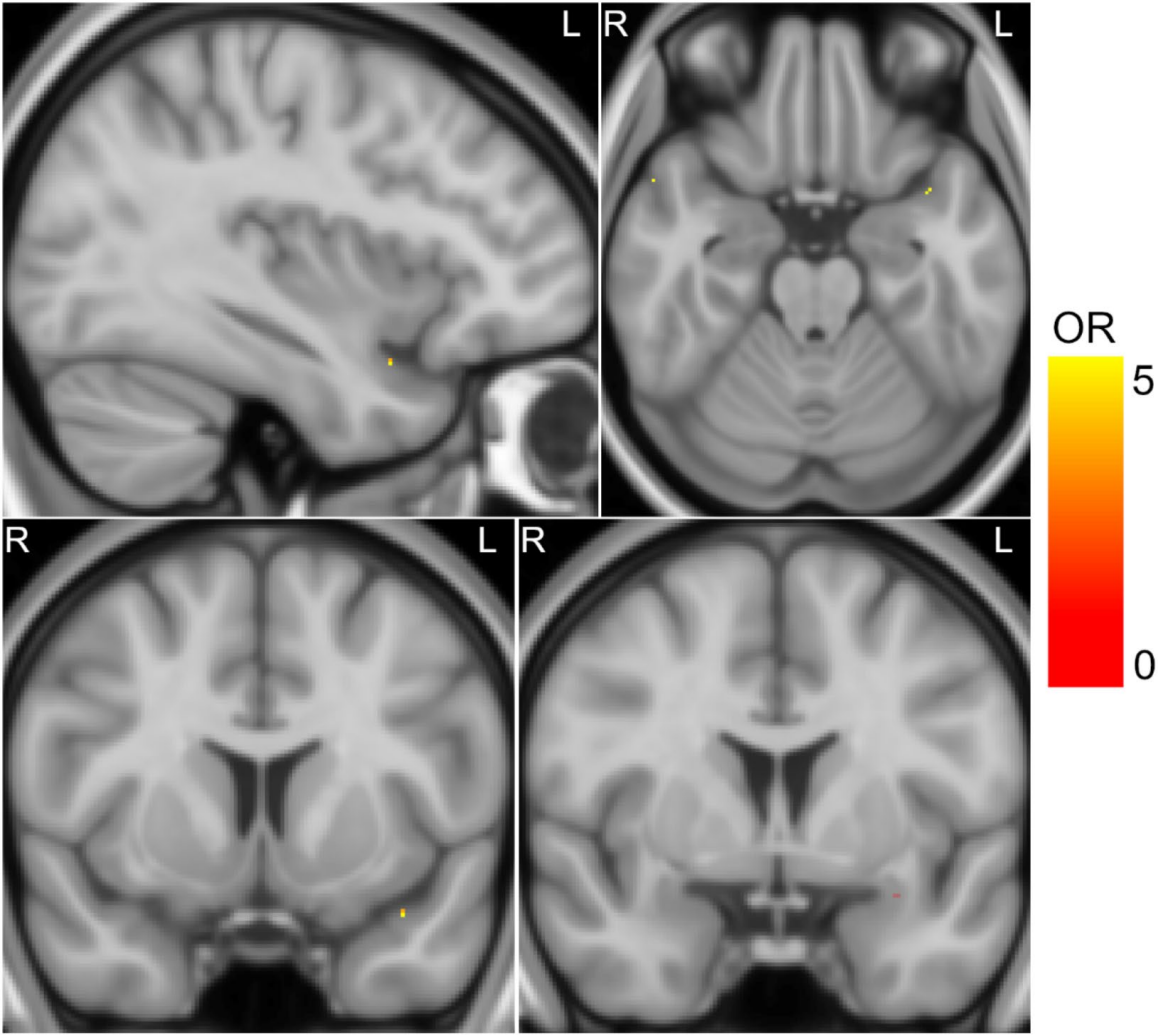
Voxelwise odds-ratios for ILAE-I outcomes obtained from voxel-based lesion-outcome mapping using logistic regression. Depicted results are thresholded at an uncorrected p-value of 0.001 and should therefore not be interpreted as statistically significant. Few voxels immediately rostral to the left anterior piriform cortex appear to show increased odds for ILAE-I outcomes, while few temporal piriform cortex voxels showed decreased odds (bottom right panel, OR = 0.26 to 0.29). Background image: MNI152 template

Finally, lesion-outcome mapping was performed using a voxelwise logistic regression framework to assess associations between regional resections and ILAE class I outcomes. This analysis included all 305 subjects from the IDEAS cohort and was independent from any prespecified definitions of piriform cortex boundaries. No associations were found in either the cohort with right (*n* = 135 patients) or left (*n* = 170 patients) TLE. Figure 4 descriptively depicts voxelwise odds ratios for seizure freedom passing an uncorrected threshold of *p* < 0.001. Clusters located in piriform cortex were restricted to the left hemisphere, with lower odds for seizure freedom in cases where certain voxels within left temporal piriform cortex had been resected (notably uncorrected).

##### Voxel-based lesion-outcome mapping in hippocampal sclerosis only

An additional voxel-based lesion-outcome analysis was restricted to the subgroup of 205 subjects with HS to test for potential disease-specific effects in a cohort with uniform surgical approaches (all ATLR). Here, resection zones of patients with right ATLR were mapped to the left hemisphere of a symmetric template to boost the number of observations and statistical power. No significant associations with ILAE I outcomes were detected.

## Discussion

This study tested whether the resection extent in investigator-independent, connectivity-based piriform cortex subregions is linked to ILAE I outcomes. Compared with prior volumetric studies that utilized manual labeling, our approach was investigator-independent, specific to piriform cortex (excluding mesial amygdala), and captured resections extending beyond the limen insulae [22–25].

In summary, no significant relationship between resected temporal lobe subregion volumes and postoperative ILAE outcomes was seen. Extensive additional analyses in a validation cohort, including voxel-based lesion-outcome mapping, corroborated this lack of an association.

In the discovery cohort, piriform cortex resection—defined either by manual segmentation or connectivity-based parcellation—was not associated with seizure freedom. Notably, resections within the manually segmented piriform cortex were smaller than 50% in most cases (Fig. 3). Prior work reported higher seizure-freedom probabilities with piriform resections > 50% [22], and only 8.5% when < 50% was resected. We did not observe this lack of seizure freedom in patients with smaller resections (43% seizure freedom (ILAE I) with < 50% resections). Nonetheless, overall smaller resection volumes and predominance of SAHE as the surgical approach may have contributed to negative findings in our discovery cohort.

Others have reported associations between temporal piriform cortex resections and seizure freedom after SAHE [24]. Here, temporal piriform cortex resections of ≥ 27% were linked to higher odds of seizure freedom. In our discovery cohort, frontal piriform resections were negligible, while temporal piriform cortex resections were more extensive (Fig. 3) and in line with these previous reports.

To address potential bias associated with the small, heterogeneous and SAHE-predominant discovery cohort, we conducted extensive validation analyses in a large independent dataset. A substantial, separately analyzed subgroup of this cohort had similar characteristics (all HS and ATLR) to the cohort in which piriform cortex–outcome associations were first reported [22]. However, no link between piriform cortex resections and outcome was detected. Voxelwise lesion–outcome mapping using logistic regression likewise revealed no spatial pattern of resection associated with seizure freedom.

Our results are therefore not in line with several previous studies [22–25]. However, negative replications have been reported. In a cohort of 50 children with TLE, no association between piriform cortex resection volumes and seizure freedom was observed [26]. Only a sub-analysis of 19 subjects with hippocampal atrophy showed a significant association limited to temporal piriform cortex resection volumes. Consistent with a more limited role of the piriform cortex in some patients with TLE, an intracranial EEG study [(N. P. [27]) *n* = 22 patients with TLE] showed only scarce involvement of the piriform cortex in epileptogenic networks (seizure onset in *n* = 1 with olfactory aura, early involvement in *n* = 2). Therefore, the assumption that the piriform cortex might have a common, major role in epileptogenic networks in patients with TLE should be viewed with caution.

## Limitations

The primary limitations of this study are the small sample size and etiological heterogeneity of the discovery cohort. Because this cohort was small, separate outcome analyses by etiology were not feasible. Outcomes after temporal lobe surgery can vary by pathology; for example, resection of discrete epileptogenic lesions such as long-term epilepsy-associated tumors may be associated with more favorable outcomes than TLE surgery overall [6, 8, 53]. The predominant surgical approach was SAHE, with smaller overall piriform cortex resections compared to previous cohorts with predominantly ATLR [22]. To address these limitations and assess the robustness of our findings, we performed validation analyses in a large independent cohort (*n* = 305), which produced consistent results.

Manual segmentation of piriform cortices was not conducted in the validation cohort. However, the piriform cortex outside the amygdala was captured using the registered connectivity-based parcellation. We additionally performed voxelwise lesion–outcome mapping, which is independent of regional definitions and corroborated the absence of an association with seizure freedom. Postoperative seizure control decreases with the duration of the follow-up period, which varied across patients [3, 54]. While the sample size of the discovery cohort did not allow for subgroup analyses, we provided a supplementary analysis of a homogeneous subpopulation of the IDEAS cohort with a fixed follow-up interval, where results remained unchanged.

## Conclusion

We found no evidence of an association between the extent of piriform cortex resection and seizure freedom in two independent cohorts, using volumetry and voxel-based lesion outcome mapping. These findings, which were robust across different definitions of piriform cortex, do not support a substantial role for piriform cortex resections in temporal lobe epilepsy surgery. This contrasts with previous reports. Surgical decision-making in TLE remains a nuanced multidisciplinary process, and the decision for piriform cortex resection should be made on an individualized basis.

## Supplementary Information

Below is the link to the electronic supplementary material.

Supplementary file1 (DOCX 2201 KB)

## Data Availability

Data from the discovery cohort is not available for reasons of data protection. The validation cohort is freely available under https://openneuro.org/datasets/ds005602/versions/1.0.0. Code for data analyses is available on request.
