# Supplementary material for: No link between piriform cortex subregion resection and seizure freedom in two cohorts with temporal lobe epilepsy
Source: J Neurol. 2026 May 22;273(6):335. doi: 10.1007/s00415-026-13850-w (PMC13197268; doi:10.1007/s00415-026-13850-w)
Supplement: Supplementary file 1 — Supplementary file1 (DOCX 2201 KB) [file 415_2026_13850_MOESM1_ESM.docx]

*No link between piriform cortex subregion resection and seizure freedom in two cohorts with temporal lobe epilepsy-* **Supplementary Material**

**1. Hippocampal subfield analysis in the discovery cohort**

| **ROI** | **t-value** | **Cohen’s_d** | **P (FDR)** |
| --- | --- | --- | --- |
| **parasubiculum** | -0.09 | -0.04 | 0.9723 |
| **HATA** | -2.00 | -0.73 | 0.1229 |
| **fimbria** | -1.97 | -0.66 | 0.1043 |
| **HP_tail** | -1.71 | -0.56 | 0.0514 |
| **presubiculum-head** | -1.50 | -0.60 | 0.1907 |
| **presubiculum-body** | -2.56 | -0.92 | 0.0539 |
| **subiculum-head** | -1.09 | -0.43 | 0.3556 |
| **subiculum-body** | -2.10 | -0.79 | 0.1134 |
| **CA1-head** | -1.67 | -0.65 | 0.1576 |
| **CA1-body** | -3.39 | -1.24 | **0.0154** |
| **CA3-head** | -1.85 | -0.72 | 0.1539 |
| **CA3-body** | -4.27 | -1.45 | **0.0062** |
| **CA4-head** | -1.71 | -0.68 | 0.1576 |
| **CA4-body** | -3.78 | -1.31 | **0.0071** |
| **GC-ML-DG-head** | -2.10 | -0.84 | 0.1134 |
| **GC-ML-DG-body** | -3.78 | -1.33 | **0.0071** |
| **molecular_layer_HP-head** | -1.82 | -0.73 | 0.1539 |
| **molecular_layer_HP-body** | -4.15 | -1.46 | **0.0069** |
| **Lateral-nucleus** | 0.38 | 0.14 | 0.8021 |
| **Basal-nucleus** | -0.93 | -0.34 | 0.4256 |
| **Central-nucleus** | -1.68 | -0.57 | 0.1576 |
| **Medial-nucleus** | -0.70 | -0.23 | 1.0000 |
| **Cortical-nucleus** | -2.28 | -0.76 | 0.1030 |
| **Accessory-Basal-nucleus** | -1.71 | -0.62 | 0.1576 |
| **Corticoamygdaloid-transition** | -1.59 | -0.60 | 0.1718 |
| **Paralaminar-nucleus** | 0.08 | 0.03 | 0.9723 |

**Table S1**. Association of hippocampal and amygdala subfield resections with ILAE I outcomes. Significant associations after FDR corrections are highlighted in bold. Only regions within the hippocampal body or tail remained associated with negative ILAE I outcomes. ROI = region of interest.

**2. Volumetric analysis- IDEAS cohort without hippocampal sclerosis**

A second sub-cohort from the IDEAS dataset without hippocampal sclerosis (n=100) was analyzed to examine whether regional resection volumes are associated with ILAE I outcomes in non-HS TLE. No significant association was detected (Table S2).

|  | **T-statistic** | **p-value** | **Cohen’s d** | **p (FDR)** |
| --- | --- | --- | --- | --- |
| **Frontal PC** | -1.34 | 0.33 | -0.41 | 0.5 |
| **Dorsotemporal PC** | NaN | NaN | NaN | 1 |
| **Anterior / temporal PC** | -1.24 | 0.22 | -0.28 | 0.5 |
| **Whole PC** | -1.28 | 0.2 | -0.28 | 0.5 |
| **Hippocampus** | -0.9 | 0.37 | -0.19 | 0.5 |
| **Entorhinal cortex** | -0.59 | 0.56 | -0.12 | 0.64 |
| **Amygdala** | -1.08 | 0.28 | -0.22 | 0.5 |
| **Overall resection** | -1.75 | 0.08 | -0.37 | 0.5 |

**Table S2**. Independent samples t-test for associations between resected proportions of temporal lobe subregions and ILAE class I outcomes. PC= piriform cortex

**3. Subfield analysis: IDEAS cohort**

No association between the EOR in subfields of the hippocampus or the amygdala with postoperative outcomes was detected. Tables S3 and S4 show FDR corrected results from multiple univariate testing in the cohort with (Table S3) and without HS (Table S4).

| **N=205 patients with HS (IDEAS)** | **T-statistic** | **p-value** | **Cohen’s d** | **p (FDR)** |
| --- | --- | --- | --- | --- |
| **Parasubiculum** | -1.02 | 0.31 | -0.14 | 0.86 |
| **HATA** | 0.38 | 0.7 | 0.05 | 0.86 |
| **Fimbria** | 0.63 | 0.55 | 0.09 | 0.86 |
| **Hippocampal Fissure** | -0.12 | 0.91 | -0.02 | 0.98 |
| **Hippocampal Tail** | 0.66 | 0.61 | 0.09 | 0.86 |
| **Presubiculum Head** | -0.78 | 0.46 | -0.1 | 0.86 |
| **Presubiculum Body** | 0.02 | 0.98 | 0.003 | 0.98 |
| **Subiculum Head** | -0.67 | 0.52 | -0.09 | 0.86 |
| **Subiculum Body** | -0.41 | 0.68 | -0.06 | 0.86 |
| **CA1-head** | -0.79 | 0.44 | -0.1 | 0.86 |
| **CA1-body** | -1.06 | 0.29 | -0.15 | 0.86 |
| **CA3-head** | 1.06 | 0.29 | 1.6 | 0.86 |
| **CA3-body** | 0.52 | 0.61 | 0.07 | 0.86 |
| **CA4-head** | 0.99 | 0.33 | 0.14 | 0.86 |
| **CA4-body** | 0.46 | 0.64 | 0.07 | 0.86 |
| **GC-ML-DG-head** | 0.63 | 0.53 | 0.09 | 0.86 |
| **GC-ML-DG-body** | 0.6 | 0.55 | 0.09 | 0.86 |
| **molecular_layer_HP-head** | -0.11 | 0.91 | 0.02 | 0.98 |
| **molecular_layer_HP-body** | 0.05 | 0.96 | 0.007 | 0.98 |
| **Lateral-nucleus** | -1.9 | 0.055 | -0.25 | 0.86 |
| **Basal-nucleus** | -1.02 | 0.31 | -0.14 | 0.86 |
| **Central-nucleus** | -0.4 | 0.69 | -0.056 | 0.86 |
| **Cortical-nucleus** | 0.81 | 0.42 | 0.11 | 0.86 |
| **Accessory-Basal-nucleus** | -0.84 | 0.4 | -0.12 | 0.86 |
| **Corticoamygdaloid-transition** | -0.18 | 0.86 | -0.03 | 0.98 |
| **Paralaminar-nucleus** | -1.32 | 0.19 | -0.18 | 0.86 |

**Table S3**. IDEAS cohort, subgroup with hippocampal sclerosis (n=205). Independent samples t-test for associations between resected proportions of ipsilateral hippocampal and amygdala subfields and ILAE class I outcomes.

| **N=100 patients without HS (IDEAS)** | **T-statistic** | **p-value** | **Cohen’s d** | **p (FDR)** |
| --- | --- | --- | --- | --- |
| **Parasubiculum** | -1.16 | 0.25 | -0.24 | 0.45 |
| **Fimbria** | -0.74 | 0.47 | -0.17 | 0.56 |
| **Hippocampal Fissure** | -1.22 | 0.22 | -0.25 | 0.45 |
| **Hippocampal Tail** | 1.27 | 0.17 | 0.19 | 0.45 |
| **Presubiculum Head** | -1.3 | 0.2 | -0.27 | 0.45 |
| **Presubiculum Body** | -0.95 | 0.35 | -0.19 | 0.46 |
| **Subiculum Head** | -1.72 | 0.1 | -0.35 | 0.45 |
| **Subiculum Body** | -1.01 | 0.31 | -0.22 | 0.46 |
| **CA1-head** | -1.37 | 0.17 | -0.28 | 0.45 |
| **CA1-body** | -0.95 | 0.34 | -0.21 | 0.46 |
| **CA3-head** | -0.67 | 0.5 | -0.14 | 0.57 |
| **CA3-body** | -0.48 | 0.64 | -0.1 | 0.69 |
| **CA4-head** | -1.18 | 0.24 | -0.25 | 0.45 |
| **CA4-body** | -0.82 | 0.41 | -0.18 | 0.51 |
| **GC-ML-DG-head** | -1.22 | 0.22 | -0.26 | 0.45 |
| **GC-ML-DG-body** | -1.11 | 0.27 | -0.24 | 0.45 |
| **molecular_layer_HP-head** | -1.24 | 0.22 | -0.26 | 0.45 |
| **molecular_layer_HP-body** | -0.97 | 0.34 | -0.21 | 0.46 |
| **Lateral-nucleus** | -1.2 | 0.23 | -0.24 | 0.45 |
| **Basal-nucleus** | -1.3 | 0.2 | -0.26 | 0.45 |
| **Central-nucleus** | 0.19 | 0.85 | 0.04 | 0.85 |
| **Cortical-nucleus** | -0.2 | 0.84 | -0.04 | 0.85 |
| **Accessory-Basal-nucleus** | -1.12 | 0.27 | -0.23 | 0.45 |
| **Corticoamygdaloid-transition** | -1.47 | 0.15 | -0.31 | 0.45 |
| **Paralaminar-nucleus** | -1.49 | 0.14 | -0.3 | 0.45 |

**Table S4**. IDEAS cohort, subgroup without hippocampal sclerosis (n=100). Independent samples t-test for associations between resected proportions of ipsilateral hippocampal and amygdala subfields and ILAE class I outcomes.

**4. Analyses at fixed follow-up timepoints (IDEAS cohort)**

To account for potential variability in surgical outcomes due to variance in postoperative follow-up periods, an additional analysis was conducted in subjects with a fixed follow-up interval of 3 years. The subgroup of 205 patients with HS and ATLR from the ideas cohort was filtered for subjects with available outcome data at postoperative year three (n=172). We conducted the same volumetric analyses as reported in the main analysis, with outcomes at year three as the independent variable. Results remained unchanged (Table S4.).

|  | **T-statistic** | **p-value** | **Cohen’s d** | **p (FDR)** |
| --- | --- | --- | --- | --- |
| **Frontal PC** | 0.63 | 0.74 | 0.1 | 0.97 |
| **Dorsotemporal PC** | -0.26 | 0.79 | -0.03 | 0.97 |
| **Anterior / temporal PC** | -0.7 | 0.48 | -0.1 | 0.97 |
| **Whole PC** | -0.64 | 0.52 | -0.09 | 0.97 |
| **Hippocampus** | 0.14 | 0.88 | 0.02 | 0.97 |
| **Entorhinal cortex** | 0.03 | 0.97 | 0 | 0.97 |
| **Amygdala** | 0.24 | 0.8 | 0.04 | 0.97 |
| **Overall resection** | 0.19 | 0.8 | 0.03 | 0.97 |

**Table S5.** Independent samples t-test for associations between resected proportions of temporal lobe subregions and ILAE class I outcomes. Analysis restricted to patients from the IDEAS cohort with HS at a follow-up interval of 3 years. PC= piriform cortex

**5. Representative illustration of registered piriform cortex ROI – IDEAS cohort**

The following figures illustrate registered connectivity-based piriform cortex ROI in three representative subjects from the IDEAS cohort. Note that this connectivity-based parcellation had been produced based on diffusion MRI and in a different cohort (cf. Methods section and (Zahnert et al., 2024)).


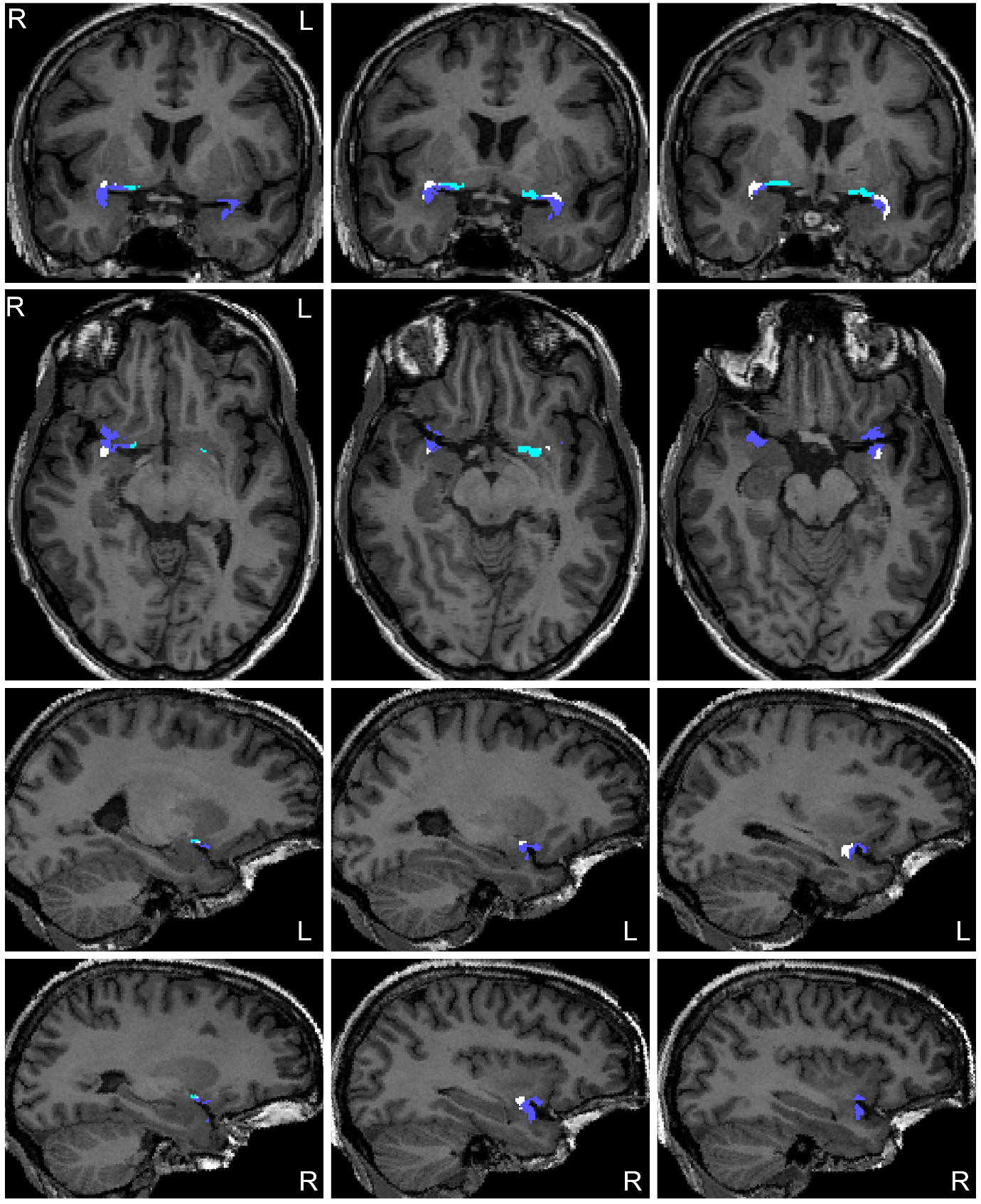


**Figure S5**. Registered piriform cortex parcellation in native space; subject 005, IDEAS dataset. Coronal slices progress from anterior to posterior, axial slices progress from dorsal to ventral, and sagittal slices progress from medial to lateral. Blue = anterior PC; white = dorsal temporal PC; Cyan = frontal PC.

**References**

Zahnert, F., Kleinholdermann, U., Belke, M., Keil, B., Menzler, K., Pedrosa, D.J., Timmermann, L., Kircher, T., Nenadić, I., Knake, S., 2024. The connectivity-based architecture of the human piriform cortex. NeuroImage 297, 120747. https://doi.org/10.1016/j.neuroimage.2024.120747
